# Optimization design of railway logistics center layout based on mobile cloud edge computing

**DOI:** 10.7717/peerj-cs.1298

**Published:** 2023-04-20

**Authors:** Xiaoling Zhang

**Affiliations:** Transportation Management College, Zhengzhou Railway Vocational and Technical College, Zhengzhou, Henan, China

**Keywords:** Cloud computing, Edge calculation, Railway logistics center, Unloading strategy

## Abstract

With the development of the economy, the importance of railway freight transportation has become essential. The efficiency of a railway logistics center depends on the types, quantities, information exchange, and layout optimization. Edge collaboration technology can consider the advantages of cloud computing’s rich computing storage resources and low latency. It can also provide additional computing power and real-time requirements for intelligent railway logistics construction. However, the cloud-side collaboration technology will introduce the wireless communication delay between the mobile terminal and the edge computing server. We designed a two-tier unloading strategy algorithm and solved the optimization problem by determining the unloading decision of each task. The cost of every task is calculated in the onboard device calculation, vehicular edge computing (VEC), and cloud computing server calculation. Simulation results show that the proposed method can save about 40% time delay compared to other unloading strategies.

## Introduction

With the development of the infrastructure and economy, railway transportation is becoming more intelligent, and the new infrastructure of railway logistics centers is speeding up (Lu et al., 2019; Hassan, Alvin Yau & Wu, 2019). Under the development policy of new infrastructure, the new network infrastructure based on 5G will provide ubiquitous connection service in the future, and the intelligent data infrastructure based on central cloud and edge cloud will provide computing power support and realize the universal computing power to promote the intelligent development of various vertical industries.

It is urgent and essential to build high-performance ubiquitous computing power, improve the intelligence level of railway equipment and realize dynamic aggregation and deep mining for effective utilization of various application data under the intelligent railway. However, the computing power of railway vehicle-mounted or special mobile terminal equipment is weak, and the intelligence level still has much room for improvement. With the policy support of new infrastructure, the introduction of cloud computing (Alam, 2020) and edge computing technology (Cao et al., 2020) will become an inevitable means to achieve the goal of the intelligent railway. Edge computing is a distributed platform that integrates network, computing, storage, and application processing capabilities at the network’s edge near the object or data source to provide intelligent services nearby. Cloud computing breaks up massive data processing programs into countless small programs, which are then processed and analyzed by servers and sent back to the user. Compared with onboard railway equipment or dedicated mobile terminals, the infrastructure edge cloud in the edge computing mode has more substantial computing power. It can perform complex computing tasks such as Big data analysis (Allam & Dhunny, 2019), image recognition (Pak & Kim, 2017), *etc.*, and the computing time is shorter, and the efficiency is higher. The terminal can unload the data that need to be analyzed and processed to the edge and central cloud through cloud edge computing. In this way, the intelligence level of railway equipment can be improved. In addition, the edge cloud and the main cloud can also be used as convergence nodes of multivariate data to meet the needs of data fusion analysis. Therefore, the collaborative computing of cloud edge and terminal is an inevitable mode for developing intelligent railways and an inevitable means to improve the intellectual level of railway equipment.

However, cloud computing and edge computing technologies have their advantages and disadvantages. Generally, cloud computing is rich in computing, storage, and network resources. Data centers of banks and large enterprises will use cloud computing technology to virtualize physical resources to dynamically and flexibly allocate resources and improve operational efficiency. However, the deployment of the central cloud is concentrated, its location is far away from the end users, and the end-to-end delay is long. However, many application scenarios are sensitive to time delays, *e.g.*, augmented reality (AR)/virtual reality (VR), car networking, and other applications. They can use mobile edge computing closer to the user side and ensure the application time delay requirements while making up for local devices’ lack of computing power through computing offload technology (Mach & Becvar, 2017; Mao et al., 2017; Taleb et al., 2017). Compared with the infrastructure of cloud computing mode, edge cloud can provide fewer resources and only serve some users. There are still crucial problems that need to be studied and solved.

This article proposes a two-layer task unloading strategy based on cloud-edge collaboration. The algorithm calculates the cost of the task in onboard device computing. It reserves the unloading decision that can get less cost to the subsequent task decision. Compared with other unloading strategies, the proposed method can effectively improve efficiency by 40%, which has a specific contribution to constructing the intelligent railway.

## Related Work

Combining cloud and edge computing can optimize execution efficiency and system power consumption. The related research on collaborative computing mainly focuses on the collaborative computing of cloud computing and edge computing (Tong et al., 2022), with the optimization objectives of completion time, energy consumption, and the weighted sum of time and energy. There are already several methods for the synergy between cloud and edge computing. In Wu et al. (2018), the authors proposed a novel heuristic method to balance the load among edge servers to minimize the overall response time. It also optimizes the load among edge servers by jointly scheduling requests and services. The results show that the algorithm’s computational complexity is low, and the corresponding overall time can be shortened. Ren et al. (2019) aimed at the partial unloading problem in the wireless transmission mode of time division multiple access to optimize the weighted sum of all users’ time delays, and a related problem model was built. This article divided it into two independent subproblems: optimizing transmission and calculation time. The former can get the minimum value by related mathematical derivation, while the KKT condition can solve the latter. Souza Vitor Barbosa et al. (2016) analyzed the QoS-aware service allocation problem under cloud-cloud hybrid architecture. The solution to this problem can meet the capacity requirements and minimize the service delay. Yang et al. (2022) considered computing resources and bandwidth resources together, aiming at reducing the execution delay and the weighted sum of energy consumption of all users, and proposes an asynchronous deep reinforcement learning algorithm under cloud-edge collaboration to make relevant migration decisions. To meet the requirements of big data scenarios and the dynamic changes of the environment under edge nodes, the algorithm considers the computing power of both cloud computing and edge computing and can adaptively adjust the migration strategy. Simulation results show that this algorithm can obtain the final migration strategy of the approximate greedy algorithm with the lowest computational cost. For tasks related to each other,  Liu et al. (2019) solved the scheduling problem that depends on the placement of tasks and on-demand function configuration on the server to minimize the completion time of application programs. Specifically, a new approximate algorithm is proposed for fixing the configuration on each edge server, which can effectively find the best task placement and scheduling (Elrab, Ahmed & Noaman, 2019).

However, the above research only focuses on collaborative computing and ignores that local devices still have specific computing power. To make more effective use of all computing resources and improve execution efficiency, collaborative computing among terminal devices, edge computing, and cloud computing is still the focus of research. The related research mainly focuses on the application of computing task independence. In  Caihong et al. (2020), the partial offloading problem, the offloading strategy, computing resources, transmission rate, and power allocation are considered effective and superior to other uninstallation schemes.

Cloud edge-end collaborative computing is still the focus of research to enhance the development of intelligent railways and meet business requirements. However, the related study of cloud-edge collaborative computing mainly considers the associated applications of public network scenarios (Moon et al., 2018). Still, it does not consider the related applications of railway scenarios, and the research on the scenarios of user mobility is less. In addition, when optimizing the total application completion time, it is necessary to study the unloading strategy and resource allocation mechanism of cloud edge-end collaborative computing that can adapt to this change.

## Intelligent Railway Logistics Center based on Cloud-Edge Collaboration Technology

**Figure 1:**
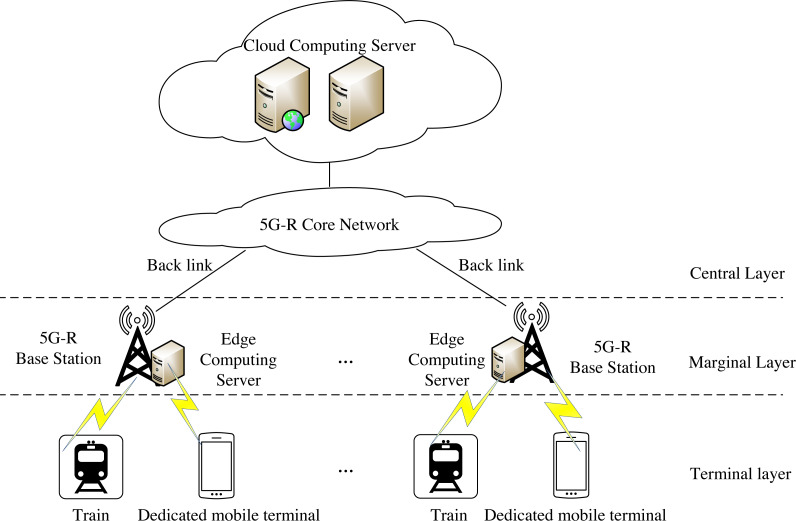
End-edge-cloud collaborative computing network architecture. Intelligent railway aims to widely apply cloud computing, Internet of Things, big data, artificial intelligence and other technologies, and build a new generation of railway transportation system through comprehensive perception and ubiquitous interconnection of railway logistics mobile equipment, fixed infrastructure and related internal and external environmental information. This article designs a computing offload algorithm under cloud-edge collaborative mode.

Intelligent railway aims to widely apply cloud computing, the Internet of Things, big data, artificial intelligence, and other technologies to build a new generation of the railway transportation system. This article designs a computing offload algorithm under cloud-edge collaborative mode. The network architecture of edge collaborative computing is shown in Fig. 1.

The terminal layer mainly comprises trains or special mobile terminals, which can generate some tasks that need to be calculated. The edge layer mainly comprises wireless access infrastructure such as base stations and edge computing servers. Generally, edge computing servers are deployed near base stations, and one edge computing server can serve multiple wireless communication cells. Wireless access infrastructure, such as base station, provides necessary communication conditions for edge computing servers and trains or dedicated mobile terminals on the terminal layer to exchange information between terminals and edge computing servers. The center layer is mainly composed of cloud computing servers with rich resources. Cloud computing servers are deployed in centralized computer rooms far away from terminals and edge computing servers, such as data centers of railway bureaus. A cloud computing server can manage multiple edge computing servers and communicate with each other through wired transmission. Under the cloud edge collaborative computing network architecture, the terminal, edge, and center layers can work together to complete a computing task. The edge computing server can make the traffic terminate locally and respond to the end users more quickly. However, the cloud computing server is far from trains or dedicated mobile terminals. Therefore, its response time is extended. It can be used as a supplement when the resources of the edge computing server are insufficient, thus alleviating the load pressure on the edge side. This architecture can effectively enhance the execution efficiency and user experience of applications. At the same time, network operators can authorize trains or dedicated mobile terminals to access their infrastructure.

### Uninstall process under cloud edge collaborative computing

**Figure 2:**
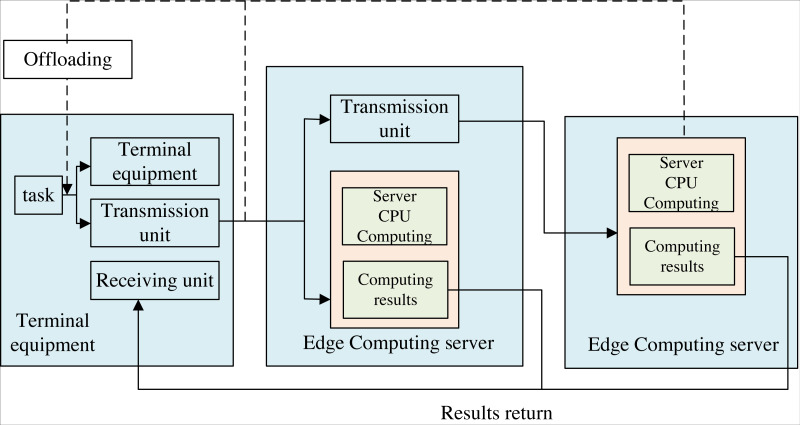
Computing offloading process under end-edge-cloud collaborative computing. The uninstallation process under cloud edge collaborative computing is shown in the figure. There are mainly two kinds of computing unload technology, which are based on edge computing and cloud computing. The former is to unload computing tasks to a cloud server with powerful resources that allows user tasks to be processed, while the latter is to unload computing tasks to an IT service environment deployed at the edge of the network for input processing. Cloud computing is deployed in a centralized manner, and resources are concentrated in a large data center. However, resources are far away from users and the latency is long. On the other hand, the deployment of edge computing is relatively dispersed and provides low latency services in a way closer to the end user, but the resources provided by edge computing are relatively limited. The uninstallation process based on cloud edge collaborative computing mainly consists of four parts: application task segmentation, uninstallation decision, calculation execution and result return.

The de-installation process under cloud-edge collaborative computing is shown in Fig. 2. There are mainly two kinds of computing unload technology: edge computing and cloud computing. The former is to offload computing tasks to a cloud server with powerful resources that allows the users’ tasks to be processed. The latter is to unload computing tasks to an IT service environment deployed at the network’s edge for input processing. Cloud computing is deployed centralized, and resources are concentrated in a large data center. However, resources are far from users, and the latency is long.

On the other hand, the deployment of edge computing is relatively dispersed and provides low-latency services closer to the end user. Still, the resources offered by edge computing are somewhat limited. The de-installation process based on cloud-edge collaborative computing mainly consists of four parts: application task segmentation, uninstallation decision, calculation execution, and results, as shown in Fig. 2.

**Figure 3:**
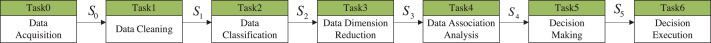
The workflow of railway logistics center information exchange. The task model of railway logistics center information exchange is shown in the figure. It includes seven steps from data collection to decision execution.

Given various information transmission, train scheduling, status report, and other tasks of the intelligent railway logistics center, the task segmentation section divides the whole application into multiple calculation tasks. It executes different unloading decisions for each calculation task once. According to different types of people, the tasks can be divided into independent applications among the tasks and the people. The model of the railway logistics center is shown in Fig. 3. It includes seven steps, from data collection to decision execution.

### Establishment of uninstall strategy problem

Firstly, establishing the state space, which reflects the information of computing resources in the cluster and the system’s current situation, is an essential basis for the decision-making behavior of the system. Therefore, the system defines the state space based on the occupied computing resources of each server in the cluster. The state space contains the occupied rack unit (RU) number of each server in the cluster. A key parameter event is also involved in the state space. The event represents the upcoming event closely related to the state transition process. The system needs to make decisions according to the events happening in the current state. To sum up, the state space defined by this system is:

(1) \begin{eqnarray*}S=\{ s{|}s=({k}_{1},{k}_{2},\ldots ,{k}_{m},\ldots ,{k}_{L},e)\} .\end{eqnarray*}

Where *k*_*m*_, *m* ∈ [1, *L*] represents the number of occupied RU of the server whose serial number is *m* in the cluster; *M* is the maximum number of RU owned by the server, and *L* is the size of the server cluster. This parameter is used to represent the usage of computing resources.

Event *e* is represented as:

(2) \begin{eqnarray*}e\in ={A}_{1},{A}_{2},\ldots ,{A}_{i},{D}_{1},{D}_{2},\ldots ,{D}_{j},\ldots ,{D}_{L}.\end{eqnarray*}

Where *A*_*i*_, *i* ∈ [1, *L*] represents whether a service uninstallation request is generated in the service range of the server whose serial number is i in the cluster; *D*_*j*_, *j* ∈ [1, *L*] represents that a calculation task is completed and a RU unit is released in server j with serial number. The combination of *D*_*j*_ and *A*_*i*_ forms event *e*.

As for the establishment of behavior space, when an event occurs, the system needs to make decisions behaviors according to the current state and events. All decision-making behaviors that can be taken in the system are included in the behavior space *A*:

(3) \begin{eqnarray*}A=-1,0,1,2,\ldots ,m,\ldots ,L.\end{eqnarray*}

For a given time, the decision system can wear and love the behavior contained in the behavior space set *A*_*s*_:

(4) \begin{eqnarray*}{A}_{s}= \left\{ \begin{array}{@{}l@{}} \displaystyle -1e\in {D}_{1},{D}_{2},\ldots ,{D}_{L}\\ \displaystyle 0,1,2,\ldots ,m,\ldots ,Le\in {A}_{1},{A}_{2},\ldots ,{A}_{L} \end{array} \right. \end{eqnarray*}

Where *A*_*s*_ =  − 1 indicates that a computing task is completed and the computing resource space is released successfully; *A*_*s*_ = *m* represents that the unloading request of the current incoming computing task is unloaded to the m server for processing. Due to the development of task decomposition technology, each incoming computing task can be regarded as a minimum computing task unit, so the system uniformly assigns a RU for processing; *A*_*s*_ = 0 indicating that the de-installation request of the current computing task is rejected

### Double-layer unloading algorithm

According to the above modeling, different unloading decisions for each task will lead to different time costs for calculating the task. Meanwhile, only when the previous task is processed can the subsequent task continue to be executed. Therefore, the following task’s unloading time depends on the last task’s decision. Because the train keeps moving in the cloud-edge collaborative computing process, the different unloading time makes the distance between the train and the base station different when the data of subsequent tasks are uploaded or sent, which leads to different communication time costs. Therefore, when optimizing the total time cost of completing the application, the unloading decision of the previous task will inevitably affect the unloading decision of the subsequent task. This article designs a serial workflow unloading algorithm suitable for cloud-edge collaborative computing mode based on this.

The pseudo-code of the algorithm is shown in Algorithm 1. Since the first task (task 0) and the last task (task N +1) must be performed on the train, this article only needs to consider unloading decisions for tasks 1 through N. Task 1 has three computing strategies: computed by onboard devices, VEC servers, or cloud computing servers. Therefore, line 3 in Algorithm 1 calculates the cost required by these three calculation methods. Based on the known unloading decisions of the previous J-1 computing tasks, lines 4 to 8 of algorithms calculate the cost of computing task j in the vehicle device, VEC server, and cloud computing server, respectively. In line 5, when the onboard device calculates task j, the time cost required by task J-1 is calculated by the onboard device, VEC server, and cloud computing server, respectively. The unloading decision with less cost is reserved for the subsequent task decision. The same goes for lines 6 and 7. Line 6 calculates the time cost of task j calculated by the VEC server, task J-1 calculated by vehicle equipment, VEC server, and cloud computing server, respectively, and retains the unloading decision that can obtain less cost for the use of subsequent task decisions. Line 7 calculates the time cost required by task j. When the cloud computing server calculates task j, task j-1 is calculated by the VEC and cloud computing servers. This process also retains the unloading decision with less cost for the subsequent task decision. Lines 9-12 calculate the time cost of task N+1 calculated by the onboard device, VEC server, and cloud computing server, respectively, and take the little cost as the final total time cost of a completed application.

**Table:** 

Algorithm 1. Two-layer offloading algorithm.
1 Initialization: ${a}_{0}={a}_{N+1}=0,{T}_{0}^{v}={T}_{N+1}^{v}={T}_{0}^{e}={T}_{N+1}^{e}=0$;
2 Input: ${T}_{n}^{v},{T}_{n}^{e},{T}_{n}^{c},{T}_{n}^{wu},{T}_{n}^{wd}$;
3 ${P}_{1}^{v}={T}_{1}^{v},{P}_{1}^{e}={T}_{0}^{u},+{T}_{1}^{e},{P}_{1}^{c}={T}_{0}^{u}+{T}_{0}^{wu}+{T}_{1}^{c}$;
4 for *j* = 2:*N* − 1 do
5 ${P}_{j}^{v}=\min \{ {P}_{j-1}^{v}+{T}_{j}^{v},{P}_{j-1}^{e}+{T}_{j-1}^{d}+{T}_{j}^{v},{P}_{j-1}^{wd}+{T}_{j-1}^{wd}+{T}_{j-1}^{d}+{T}_{j}^{v})$;
6 ${P}_{j}^{e}=\min \{ {P}_{j-1}^{v}+{T}_{j-1}^{u}+{T}_{j}^{e},{P}_{j-1}^{e}+{T}_{j}^{e},{P}_{j-1}^{c}+{T}_{j-1}^{wd}+{T}_{j}^{c})$;
7 ${P}_{j}^{c}=\min \{ {P}_{j-1}^{v}+{T}_{j-1}^{u}+{T}_{j-1}^{wu}+{T}_{j}^{c},{P}_{j-1}^{e}+{T}_{j-1}^{wu}+{T}_{j}^{c},{P}_{j-1}^{c}+{T}_{j}^{c})$;
8 end for
9 ${P}_{N}^{v}=\min \{ {P}_{N-1}^{v}+{T}_{N}^{v},{P}_{N-1}^{e}+{T}_{N-1}^{d}+{T}_{N}^{v},{P}_{N-1}^{c}+{T}_{N-1}^{wd}+{T}_{N-1}^{d}+{T}_{N}^{v})$;
10 ${P}_{N}^{e}=\min \{ {P}_{N-1}^{v}+{T}_{N-1}^{u}+{T}_{N}^{e},{P}_{N-1}^{e}+{T}_{N}^{e},{P}_{N-1}^{c}+{T}_{N-1}^{wd}+{T}_{N}^{c})$;
11 ${P}_{N}^{c}=\min \left\{ {P}_{N-1}^{v}+{T}_{N-1}^{u}+{T}_{N-1}^{wu}+{T}_{N}^{c}+{T}_{N}^{c}+{T}_{N}^{wd}+{T}_{N}^{d},{P}_{j-1}^{e}+{T}_{j-1}^{wu}+{T}_{j}^{c},{P}_{N-1}^{e}+{T}_{N-1}^{wu}+{T}_{N}^{c}+{T}_{N}^{wd}+{T}_{N}^{d},{P}_{N-1}^{c}+{T}_{N}^{c}+{T}_{N}^{wd}+{T}_{N}^{d} \right\} ;$
12 $\min {C}_{A}=\min {P}_{N}^{v},{P}_{N}^{e},{P}_{N}^{c}$;
13 output min*C*_*A*_ andcloud edge three-layer unloading strategy *A* at this cost.

## Simulation Results and Performance Analysis

### Parameter setting

This article selects three unloading strategies as the reference for performance evaluation: non-unloading strategy, that is, all tasks are executed on the vehicle-mounted equipment; Full edge server offload strategy, that is, all tasks are executed on the VEC server; and the all-cloud server uninstallation strategy. All tasks are performed on the cloud server. The settings of simulation parameters are shown in Table 1.

**Table 1:** The settings of simulation parameters. In order to evaluate the performance of the algorithm designed in this chapter, this article selects three unloading strategies as the reference for performance evaluation: non-unloading strategy, that is, all tasks are executed on the vehicle-mounted equipment; Full edge server offload strategy, that is, all tasks are executed on VEC server; and the all-cloud server uninstallation strategy. All tasks are executed on the cloud server.

Parameters	Description	Value
*S*_0_	The initial input data of the task	200 MB
*a*, *b*, *c*, *d*	The weight factors of the task	0.7, 10^7^, 10^5^, 5000
*F*_*v*_	VEC server and Vehicle-mounted equipment execution speed ratio	2.5
*R*	Cell radius	1500 m
Δ*t*	Time slot length	3.5
*R*_*w*_	Wired transmission rate	50, 70, 100 Mbps

### Total time cost comparison

**Figure 4:**
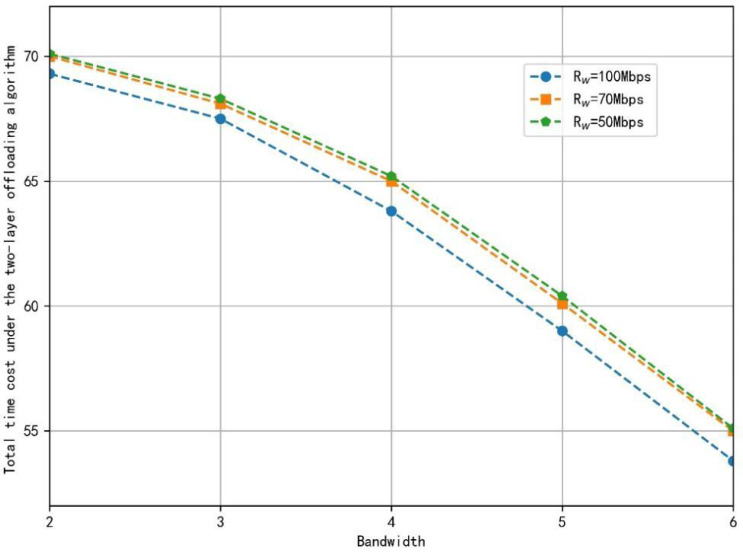
The total time cost under the two-layer offloading algorithm at different wired transmission rates.

The difference in the location of the small area where the data transmission starts in the training cell will lead to the difference in the communication distance between the base station and the train, affecting the cost of wireless transmission and the unloading decision. Therefore, this article calculates the total time cost required for the train to start its application in all small areas and takes the average of these results for subsequent performance analysis. The total time cost at different wired transmission rates is shown in Fig. 4.

**Figure 5:**
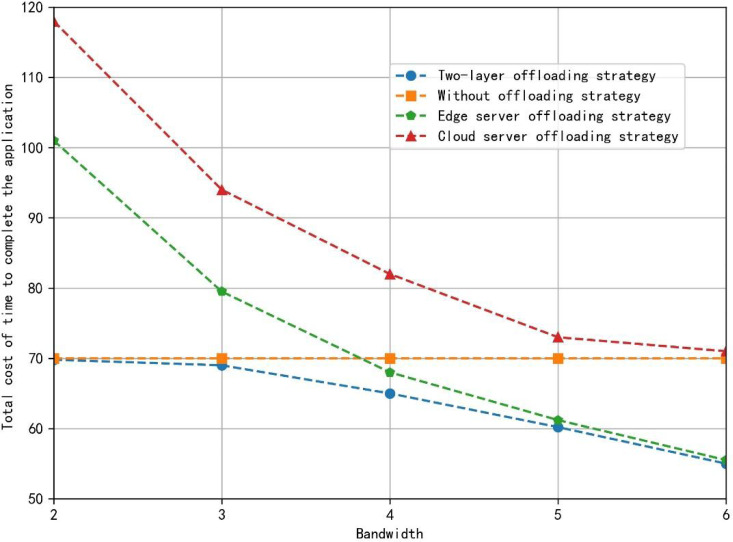
The total application completion time under different offloading strategy. The two-layer offloading algorithm always makes the total time cost of completing the application the lowest, which shows its better the performance.

Figure 5 shows the total application completion time under the different offloading strategies. The two-layer offloading algorithm always makes the total time cost of completing the application the lowest, which shows its better performance. When the bandwidth is small, due to the long communication time, the time cost saved by the substantial computing power of the VEC server and cloud computing server still can’t make up for the cost of communication time. Currently, the cloud server de-installation strategy takes the longest time. The non-de-installation and two-layer offloading strategies only need about 40% of the time cost compared with the server uninstallation strategy. In addition, the communication cost of unloading tasks decreases, and the time saved by the VEC server’s strong computing power can make up for the cost of certain communication time. Some tasks will be unloaded to the VEC server for computing. However, due to the limitation of the wired transmission rate, the task will not be continuously unloaded to the cloud computing services. When the bandwidth provided is sufficient, compared with the non-offload strategy, the full-edge server offload strategy and the two-tier offload algorithm strategy take the same time and save more time cost. Compared with the all-cloud server uninstallation strategy with the highest time cost, the proposed strategy saves about 23% of the time cost. However, the time cost of wired transmission is still the bottleneck of continued unloading to the cloud computing server, and the task will still not be unloaded to the cloud computing services.

### Time cost analysis in a fixed small area

**Figure 6:**
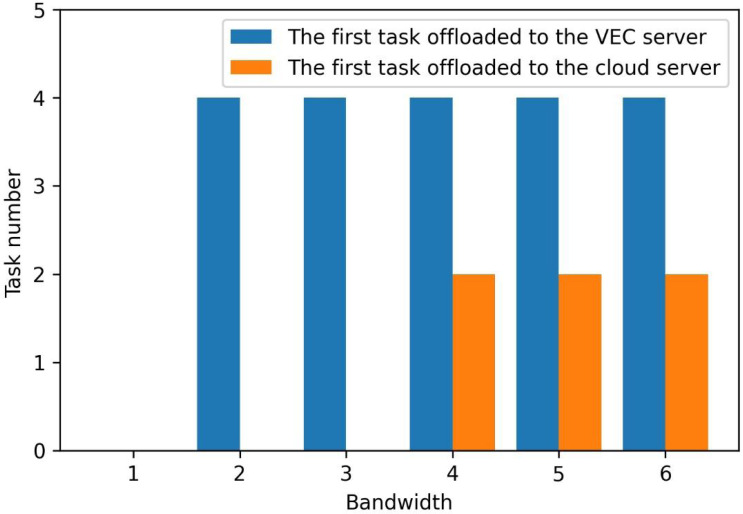
The first offloaded task at edge and cloud at the cell. The figure shows the first task to uninstall to edge servers and cloud servers when the bandwidth changes from 2MHz to 6MHz in a small area. With the increase of bandwidth, the tasks are no longer all calculated locally, but unloaded to the edge server and cloud computing server, and the first unloading task tends to happen earlier. This means that the two-layer unloading algorithm strategy can unload more tasks to the edge and cloud.

Figure 6 shows the first task to uninstall edge and cloud servers when the bandwidth changes from 2MHz to 6MHz in a small area. With the increased bandwidth, the tasks are not calculated locally but unloaded to the edge and cloud computing servers. The first unloading task tends to happen earlier. This means that the two-layer unloading algorithm strategy can unload more tasks to the edge and cloud.

**Figure 7:**
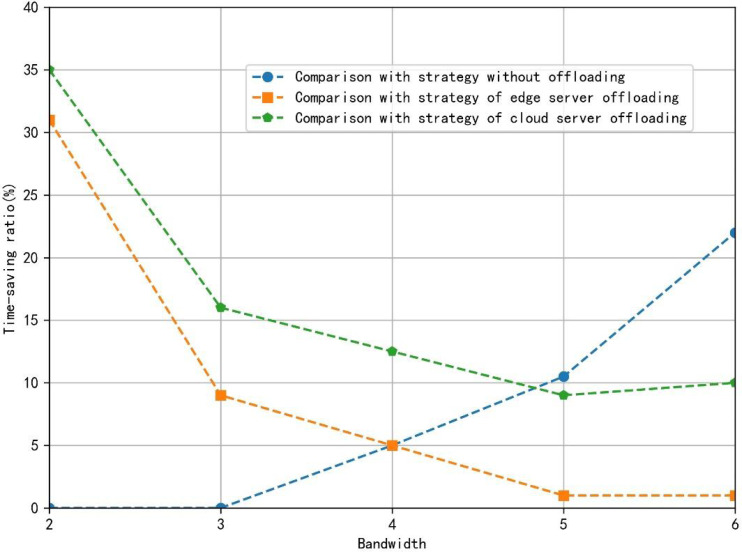
The proportion of time saved by different strategies. The figure depicts the percentage saved by the two-layer unloading algorithm strategy compared with other strategies when the bandwidth of the train changes from 2MHz to 6MHz in the fixed small area.

Figure 7 depicts the percentage saved by the two-layer unloading algorithm strategy compared with other strategies when the bandwidth of the train changes from 2MHz to 6MHz in the fixed small area. The proposed algorithm can effectively save the total time to complete the application, whether the train is at the edge of the cell or the center of the cell within the bandwidth taken by the simulation. When the bandwidth is small, compared with the full-edge server offload strategy and the full-cloud server offload strategy, the offload strategy proposed in this article saves more time because the limited bandwidth and the cost of wireless transmission time are high, so the vehicle-mounted equipment will complete more calculations. When the bandwidth is considerable, compared with the full-edge server offload strategy and the full-cloud server offload strategy, the offload strategy proposed in this article saves less time. However, compared with the non-offload strategy, the cost of saved time increases.

## Conclusion

Aiming the problem that the onboard equipment or special mobile equipment of railway logistics centers has weak computing power and needs to be improved in intelligence level. This article proposes a dual-layer task unloading strategy based on cloud-edge collaboration technology. In this algorithm, the cost of the previous task of each task is calculated respectively in the onboard device calculation, VEC server calculation and cloud computing server calculation, and the unloading decision that can obtain less cost is reserved for the subsequent task decision. The simulation results show that compared with other unloading strategies, the unloading strategy proposed in this article can save up to 40% of the end-to-end time cost. In addition, applying this technology can significantly enhance the speed of communication between equipment and improve the efficiency of railway information transmission.

Although this article considers the influence of the change of wireless transmission capacity caused by the mobility of trains, this article has not yet researched the scenario of the train switching from the area served by one VEC server to the area served by another. Therefore, the follow-up work can focus on the handover scenario of edge computing, focusing on the handover strategy and service migration strategy design.

##  Supplemental Information

10.7717/peerj-cs.1298/supp-1 Supplemental Information 1 Code

## Additional Information and Declarations

### Data Availability

The following information was supplied regarding data availability: The code is available in the Supplementary File and the data is available at GitHub: https://github.com/swinedge/eua-dataset.
