# Supplementary material for: Optimization design of railway logistics center layout based on mobile cloud edge computing
Source: PeerJ Comput Sci. 2023 Apr 20;9:e1298. doi: 10.7717/peerj-cs.1298 (PMC10280669; doi:10.7717/peerj-cs.1298)
Supplement: Supplemental Information 1 [file peerj-cs-09-1298-s001.zip › code/docs/theme/envisedge/body_postscripts.html]

{% if not embedded %}
{%- for scriptfile in script\_files %}
{%- endfor %}
{% endif %}
{# RTD hosts this file, so just load on non RTD builds #}
{% if not READTHEDOCS %}
{# I'm sorry, I don't know how to use sphinx to inject this into the static JS. #}
{% endif %}
{# STICKY NAVIGATION #}
{% if theme\_sticky\_navigation %}
{% endif %}
