# Supplementary material for: Optimization design of railway logistics center layout based on mobile cloud edge computing
Source: PeerJ Comput Sci. 2023 Apr 20;9:e1298. doi: 10.7717/peerj-cs.1298 (PMC10280669; doi:10.7717/peerj-cs.1298)
Supplement: Supplemental Information 1 [file peerj-cs-09-1298-s001.zip › code/docs/theme/envisedge/breadcrumbs.html]

{# Support for Sphinx 1.3+ page\_source\_suffix, but don't break old builds. #}
{% if page\_source\_suffix %}
{% set suffix = page\_source\_suffix %}
{% else %}
{% set suffix = source\_suffix %}
{% endif %}
{% set github\_user = 'NimbleEdge' %}
{% set github\_repo = 'EnvisEdge' %}
{% set github\_version = 'master' %}
{% set conf\_py\_path = 'docs/source' %}

- Documentation
{% for doc in parents %}
{% if doc.title == "<no title>" %}- EnvisEdge
{% else %}- {{ doc.title }}
{% endif %}
{% endfor %}
{% if title == "<no title>" %}- EnvisEdge
{% elif pagename == "search" %}- Search
{% else %}- {{ title }}
{% endif %}
{% if not pagename == "search" %}- Edit on GitHub
{% endif %}
