# Supplementary material for: Optimization design of railway logistics center layout based on mobile cloud edge computing
Source: PeerJ Comput Sci. 2023 Apr 20;9:e1298. doi: 10.7717/peerj-cs.1298 (PMC10280669; doi:10.7717/peerj-cs.1298)
Supplement: Supplemental Information 1 [file peerj-cs-09-1298-s001.zip › code/docs/theme/envisedge/build_info.html]

{%- if (build\_id and build\_url) or commit or last\_updated or last\_edited %}

{%- if build\_id and build\_url %}
{% trans build\_url=build\_url, build\_id=build\_id %}
Build
{{ build\_id }}.
{% endtrans %}
{%- elif commit %}
{% trans commit=commit %}
Revision `{{ commit }}`.
{% endtrans %}
{%- elif last\_updated %}
{% trans last\_updated=last\_updated|e %}Updated {{ last\_updated }}{% endtrans %}
{%- endif %}

{%- endif %}
