# Supplementary material for: Optimization design of railway logistics center layout based on mobile cloud edge computing
Source: PeerJ Comput Sci. 2023 Apr 20;9:e1298. doi: 10.7717/peerj-cs.1298 (PMC10280669; doi:10.7717/peerj-cs.1298)
Supplement: Supplemental Information 1 [file peerj-cs-09-1298-s001.zip › code/docs/theme/envisedge/footer.html]

{% if next or prev %}

{% if prev %}
 Previous
{% endif %}
{% if next %}
Next 
{% endif %}

{% endif %}

---

{%- if show\_copyright %}

{%- if hasdoc('copyright') %}
{% trans path=pathto('copyright'), copyright=copyright|e %}© Copyright {{ copyright }}{% endtrans %}
{%- else %}
{% trans copyright=copyright|e %}© {{ copyright }}.{% endtrans %}
{%- endif %}

{%- endif %}

{%- block extrafooter %} {% endblock %}
