# Supplementary material for: Optimization design of railway logistics center layout based on mobile cloud edge computing
Source: PeerJ Comput Sci. 2023 Apr 20;9:e1298. doi: 10.7717/peerj-cs.1298 (PMC10280669; doi:10.7717/peerj-cs.1298)
Supplement: Supplemental Information 1 [file peerj-cs-09-1298-s001.zip › code/docs/theme/envisedge/google_tag_manager.html]

 {% if target\_cloud == 'azure' %}


{% else %}


{% endif %}
