# Supplementary material for: Optimization design of railway logistics center layout based on mobile cloud edge computing
Source: PeerJ Comput Sci. 2023 Apr 20;9:e1298. doi: 10.7717/peerj-cs.1298 (PMC10280669; doi:10.7717/peerj-cs.1298)
Supplement: Supplemental Information 1 [file peerj-cs-09-1298-s001.zip › code/docs/theme/envisedge/layout.html]

{# TEMPLATE VAR SETTINGS #}
{%- set url\_root = pathto('', 1) %}
{%- if url\_root == '#' %}{% set url\_root = '' %}{% endif %}
{%- if not embedded and docstitle %}
{%- set titlesuffix = " — "|safe + docstitle|e %}
{# Add version number suffix to legacy spark documentation page titles #}
{%- if 'spark/1.6' in pagename %}
{%- set titlesuffix = ' (Spark 1.6)' ~ titlesuffix %}
{%- endif %}
{%- else %}
{%- set titlesuffix = "" %}
{%- endif %}

  


{{ metatags }}
{% block htmltitle %}
{% if title|striptags|e =="<no title>" %}
EnvisEdge 0.0.1 documentation
{% else %}
{{ title|striptags|e }}{{ titlesuffix }}
{% endif %}
{% endblock %}
{# CANONICAL #}
{# FAVICON #}
{% if favicon %}
{% endif %}
{% if s %}
{% endif %}
{# Google Tag Manager #}
{% if target\_cloud == 'azure' %}

{% else %}
{% endif %}
{# CSS #}
{# OPENSEARCH #}
{% if not embedded %}
{% if use\_opensearch %}
{% endif %}
{% endif %}
{# RTD hosts this file, so just load on non RTD builds #}
{% if not READTHEDOCS %}


{% if target\_cloud == 'azure' %}
{% endif %}
{% endif %}
{%- block linktags %}
{%- if hasdoc('about') %}
{%- endif %}
{%- if hasdoc('genindex') %}
{%- endif %}
{%- if hasdoc('search') %}
{%- endif %}
{%- if hasdoc('copyright') %}
{%- endif %}
{%- if parents %}
{%- endif %}
{%- if next %}
{%- endif %}
{%- if prev %}
{%- endif %}
{%- endblock %}
{%- block extrahead %} {% endblock %}
{# Keep modernizr in head - http://modernizr.com/docs/#installing #}

{# Google Tag Manager #}
{# Header. Includes toggle for sidebar when on thin screen #}
{% include "header.html" %}

{# SIDE NAV BAR, TOGGLES ON MOBILE #}
{% include "side\_nav.html" %}


{% include "breadcrumbs.html" %}

{% block body %}{% endblock %}

{% include "footer.html" %}

{% include "versions.html" %}
{% if not embedded %}
{%- for scriptfile in script\_files %}
{%- endfor %}
{% endif %}
{# RTD hosts this file, so just load on non RTD builds #}
{% if not READTHEDOCS %}
{# I'm sorry, I don't know how to use sphinx to inject this into the static JS. #}
{% endif %}
{# STICKY NAVIGATION #}
{% if theme\_sticky\_navigation %}
{% endif %}
{%- block footer %} {% endblock %}
{% if use\_algolia == '1' %}
{% endif %}
