# Supplementary material for: Optimization design of railway logistics center layout based on mobile cloud edge computing
Source: PeerJ Comput Sci. 2023 Apr 20;9:e1298. doi: 10.7717/peerj-cs.1298 (PMC10280669; doi:10.7717/peerj-cs.1298)
Supplement: Supplemental Information 1 [file peerj-cs-09-1298-s001.zip › code/docs/theme/envisedge/search.html]

{#
basic/search.html
~~~~~~~~~~~~~~~~~
Template for the search page.
:copyright: Copyright 2007-2013 by the Sphinx team, see AUTHORS.
:license: BSD, see LICENSE for details.
#}
{%- extends "layout.html" %}
{% set s = True %}
{% set title = \_('Search') %}
{% set script\_files = script\_files + ['\_static/searchtools.js'] %}
{% block footer %}
{# this is used when loading the search index using $.ajax fails,
such as on Chrome for documents on localhost #}
{{ super() }}
{% endblock %}
{% block body %}

{% trans %}Please activate JavaScript to enable the search
functionality.{% endtrans %}

{% if search\_performed %}

## {{ \_('Search Results') }}

{% if not search\_results %}

{{ \_('Your search did not match any documents. Please make sure that all words are spelled correctly and that you\'ve selected enough categories.') }}

{% endif %}
{% endif %}

{% if search\_results %}

{% for href, caption, context in search\_results %}- {{ caption }}

  {{ context|e }}
{% endfor %}
{% endif %}

{% endblock %}
