# Supplementary material for: Optimization design of railway logistics center layout based on mobile cloud edge computing
Source: PeerJ Comput Sci. 2023 Apr 20;9:e1298. doi: 10.7717/peerj-cs.1298 (PMC10280669; doi:10.7717/peerj-cs.1298)
Supplement: Supplemental Information 1 [file peerj-cs-09-1298-s001.zip › code/docs/theme/envisedge/side_nav.html]

{% block sidebartitle %}
{% if theme\_display\_version %}
{%- set nav\_version = version %}
{% if READTHEDOCS and current\_version %}
{%- set nav\_version = current\_version %}
{% endif %}
{% if nav\_version %}

{{ nav\_version }}

{% endif %}
{% endif %}
{% include "searchbox.html" %}
{% endblock %}

{% if not (logo and theme\_logo\_only) %}
 {{ project }}
{% endif %}
{% block menu %}
{% set toctree = toctree(maxdepth=4, collapse=theme\_collapse\_navigation, includehidden=True) %}
{% if toctree %}
{{ toctree }}
{% else %}

{{ toc }}

{% endif %}
{% endblock %}

{% include "build\_info.html" %}

Contribute
