# Supplementary material for: Optimization design of railway logistics center layout based on mobile cloud edge computing
Source: PeerJ Comput Sci. 2023 Apr 20;9:e1298. doi: 10.7717/peerj-cs.1298 (PMC10280669; doi:10.7717/peerj-cs.1298)
Supplement: Supplemental Information 1 [file peerj-cs-09-1298-s001.zip › code/docs/theme/envisedge/versions.html]

{% if READTHEDOCS %}
{# Add rst-badge after rst-versions for small badge style. #}

Read the Docs
v: {{ current\_version }}

Versions
{% for slug, url in versions %}:   {{ slug }}
{% endfor %}

Downloads
{% for type, url in downloads %}:   {{ type }}
{% endfor %}

On Read the Docs
:   Project Home
:   Builds

---

Free document hosting provided by Read the Docs.

{% endif %}
