# Supplementary figures and images for: Optimization design of railway logistics center layout based on mobile cloud edge computing
Source: PeerJ Comput Sci. 2023 Apr 20;9:e1298. doi: 10.7717/peerj-cs.1298 (PMC10280669; doi:10.7717/peerj-cs.1298)

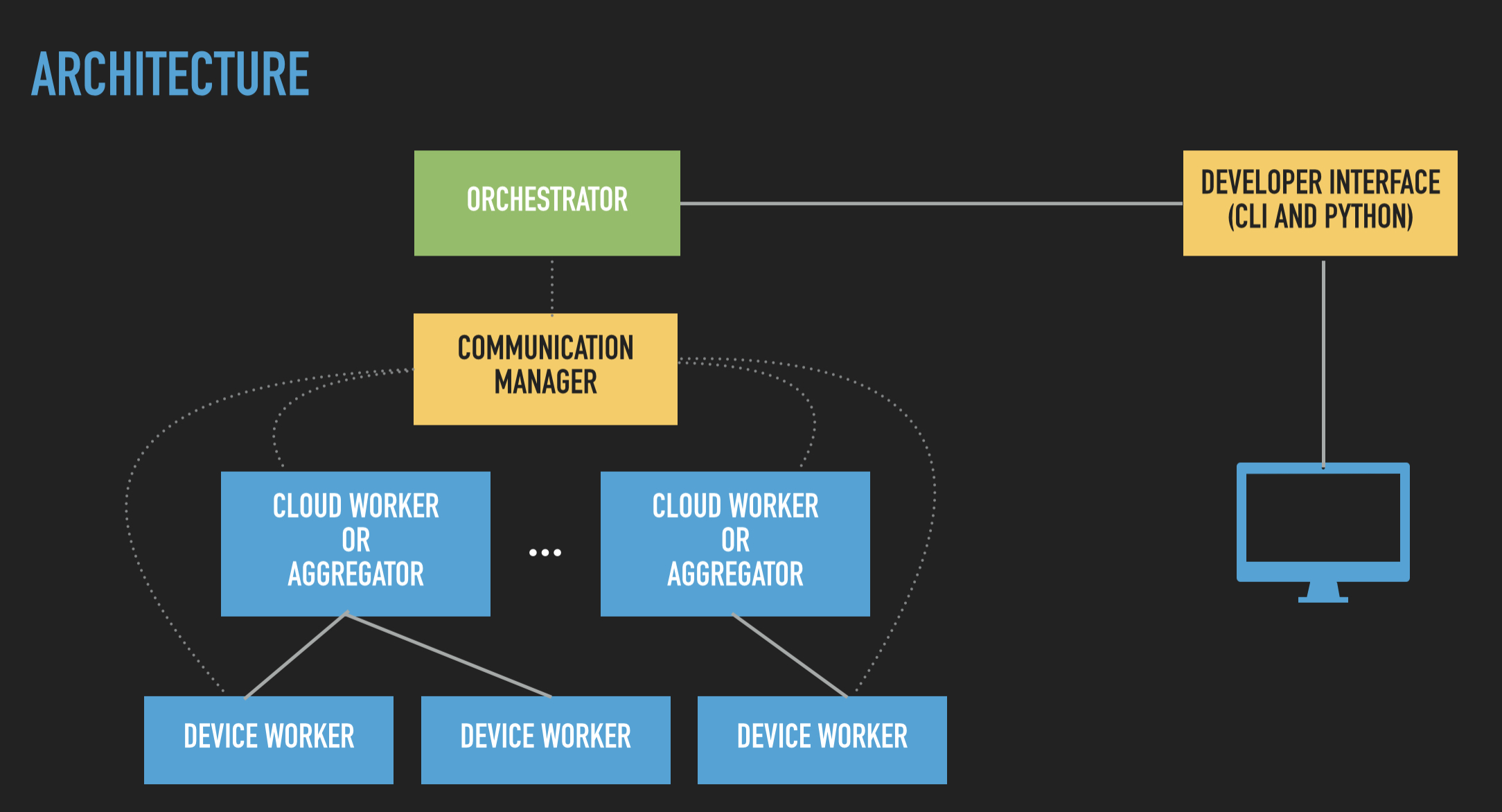

Supplement: Supplemental Information 1 [file peerj-cs-09-1298-s001.zip › code/assets/architecture.jpeg]

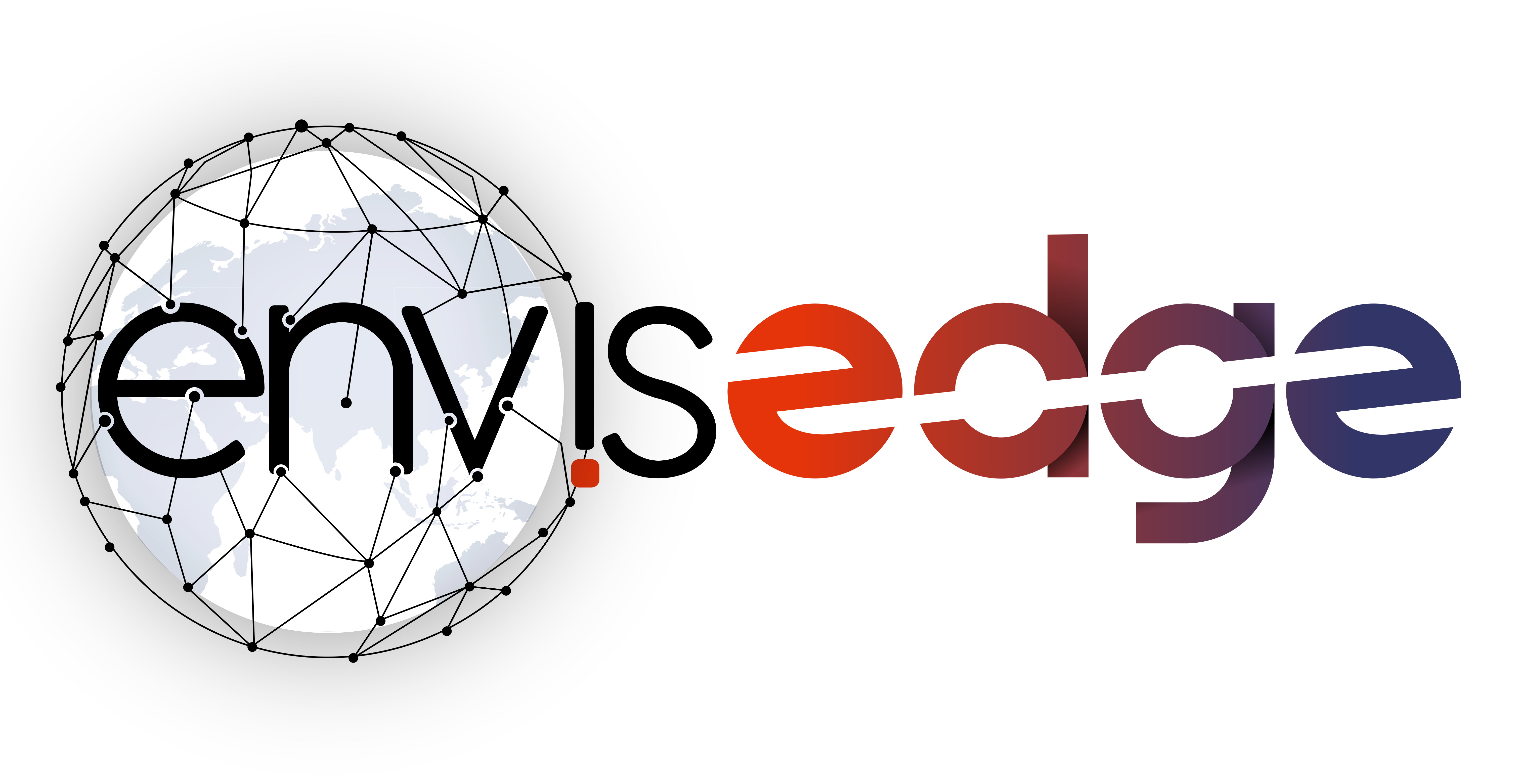

Supplement: Supplemental Information 1 [file peerj-cs-09-1298-s001.zip › code/assets/envisedge-banner-dark.png]

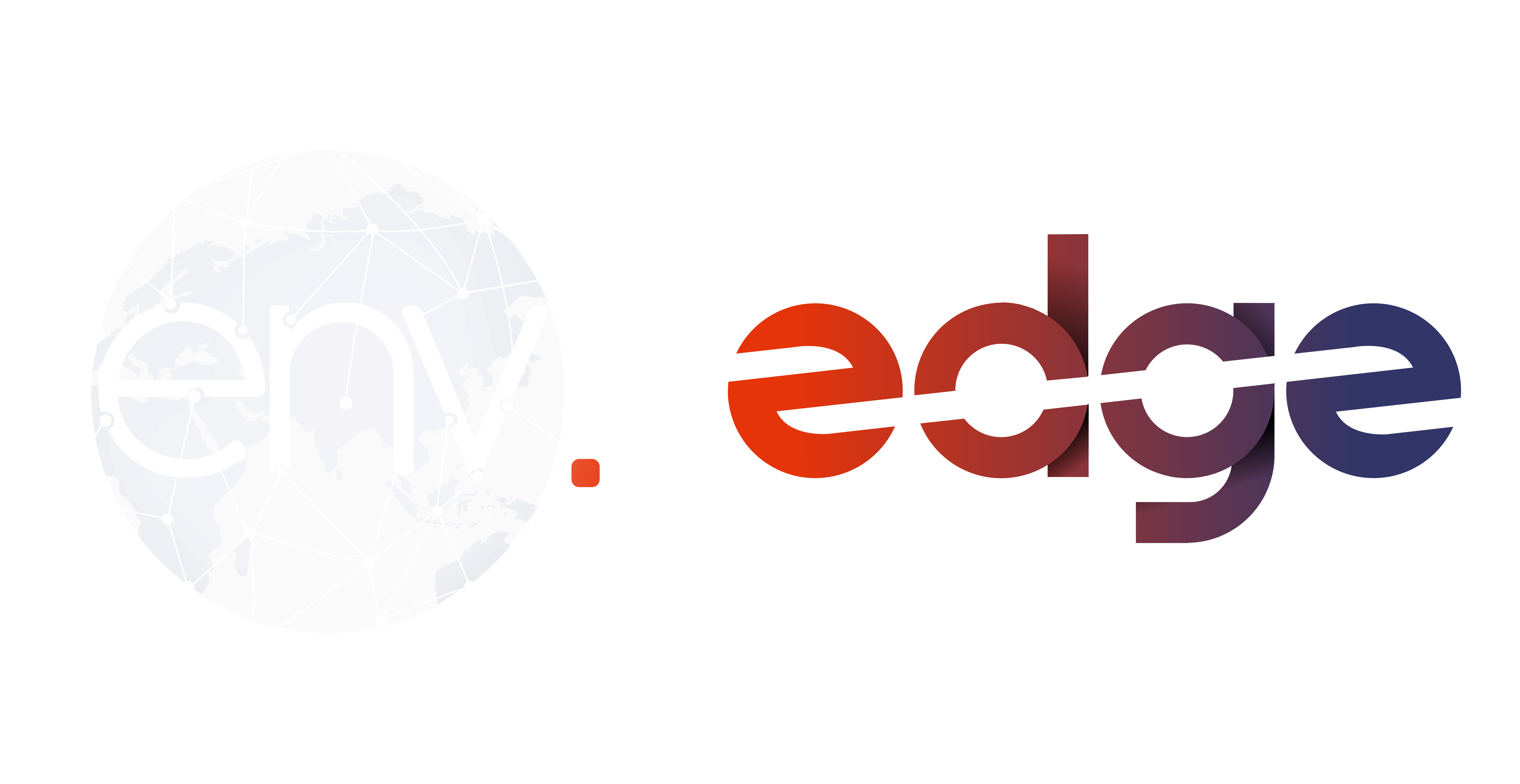

Supplement: Supplemental Information 1 [file peerj-cs-09-1298-s001.zip › code/assets/envisedge-banner-light.png]

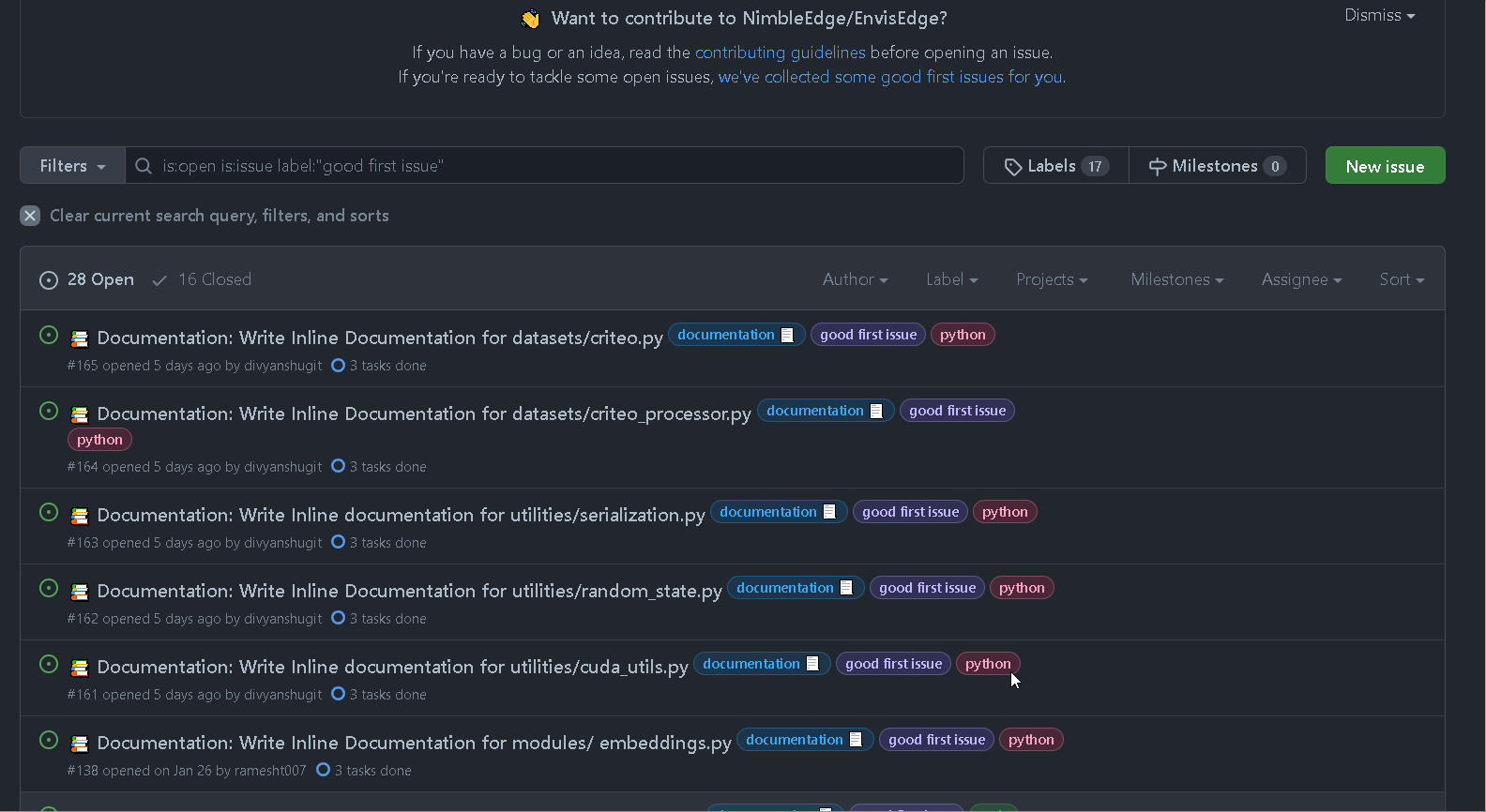

Supplement: Supplemental Information 1 [file peerj-cs-09-1298-s001.zip › code/assets/issues.gif]

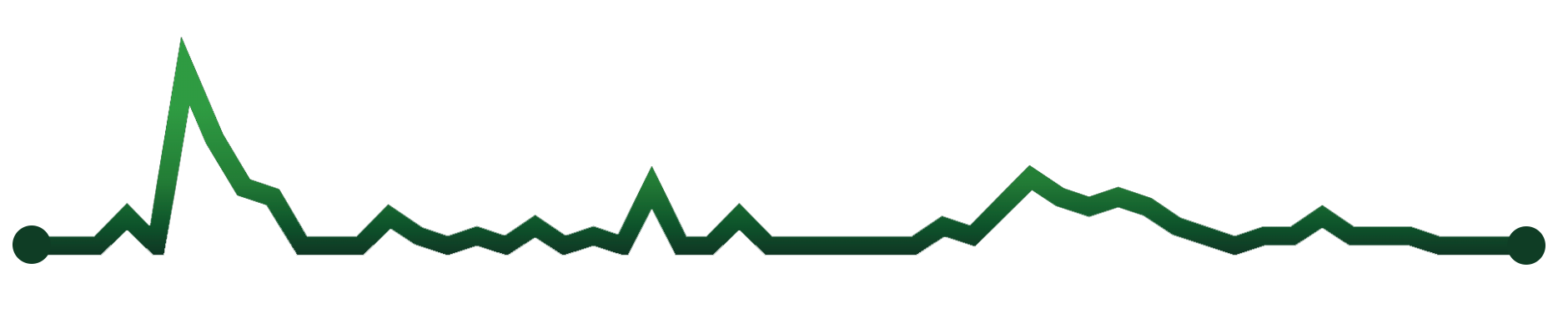

Supplement: Supplemental Information 1 [file peerj-cs-09-1298-s001.zip › code/assets/sparkline-banner.png]
